# Comparison versus reminding

**DOI:** 10.1186/s41235-016-0028-1

**Published:** 2016-12-12

**Authors:** Jonathan G. Tullis, Robert L. Goldstone

**Affiliations:** 1 Department of Educational Psychology, University of Arizona, 1430 E 2nd Street, Tucson, AZ 85721 USA; 2 Department of Psychological and Brain Sciences, Indiana University, Bloomington, IN USA

**Keywords:** False Alarm, Recognition Test, Study List, Practice Retrieval, Related Pair

## Abstract

Comparison and reminding have both been shown to support learning and transfer. Comparison is thought to support transfer because it allows learners to disregard non-matching features of superficially different episodes in order to abstract the essential structure of concepts. Remindings promote memory for the individual episodes and generalization because they prompt learners to retrieve earlier episodes during the encoding of later related episodes and to compare across episodes. Across three experiments, we compared the consequences of comparison and reminding on memory and transfer. Participants studied a sequence of related, but superficially different, proverb pairs. In the comparison condition, participants saw proverb pairs presented together and compared their meaning. In the reminding condition, participants viewed proverbs one at a time and retrieved any prior studied proverb that shared the same deep meaning as the current proverb. Experiment 1 revealed that participants in the reminding condition recalled more proverbs than those in the comparison condition. Experiment 2 showed that the mnemonic benefits of reminding persisted over a one-week retention interval. Finally, in Experiment 3, we examined the ability of participants to generalize their remembered information to new items in a task that required participants to identify unstudied proverbs that shared the same meaning as studied proverbs. Comparison led to worse discrimination between proverbs related to studied proverbs and proverbs unrelated to studied proverbs than reminding. Reminding supported better memory for individual instances and transfer to new situations than comparison.

**Electronic supplementary material:**

The online version of this article (doi:10.1186/s41235-016-0028-1) contains supplementary material, which is available to authorized users.

## Significance

Teachers and instructors aim to support their students’ long-term memories and ability to appropriately transfer learned information to new contexts. Despite this universal goal, debate persists about the most effective pedagogies to foster such retention and generalizability. Across three experiments, we compared the consequences of two prominent pedagogies (comparison and reminding) on memory and transfer. In comparison, for example, teachers may show two different chemical reactions of combustion on the screen together and have learners compare their attributes. This helps learners to recognize that combustion produces water and carbon dioxide, regardless of the reactants. In remindings, a teacher may present the different chemical equations separated in time. The later related equation may prompt learners to think back to the earlier similar equation, retrieve it from memory, and compare across equations. Both comparison and reminding have been shown to enhance learning across a variety of domains, including math, biology, and physics, but their relative merits have never been examined. We compared the benefits of reminding and comparison in order to determine the most effective pedagogical tools for educators. Across the three experiments presented here, reminding supported better memory for individual examples and enabled more appropriate transfer to new situations than comparison. Educators, then, may be able to construct educational materials and schedules to promote remindings across some kinds of related information instead of promoting explicit, simultaneous comparison of related exemplars. When confident that all students will be able to make the appropriate connections between related information, educators can prompt remindings by separating related information in time and encouraging learners to retrieve prior related examples.

## Background

Creating long-lasting and transferrable knowledge from individual episodes is important to succeeding in an ever-changing world. We must apply knowledge gained in past situations to new and different situations in order to thrive in a complex world. Making comparisons and being reminded have both been shown to create generalizable knowledge from individual episodes. Comparison has been presented as one of the most effective means of ensuring that learners generalize across instances and transfer knowledge to novel challenges (Gick & Holyoak, 1983; Loewenstein, Thompson, & Gentner, 1999). Similarly, remindings have been shown to support generalization in problem-solving and category-learning (Ross, 1984; Ross & Kennedy, 1990). In three experiments, we directly compared the consequences of comparisons and remindings on memory and generalization. In the first two experiments, we analyzed the mnemonic consequences of comparisons and remindings. In the third, we examined the consequences of comparisons and remindings on generalization and transfer. To preview, we find mnemonic and transfer benefits of remindings over comparison in all three experiments.

## Comparison

Comparison, the act of examining two like things in conjunction to assess commonalities and differences (Namy & Gentner, 2002), is thought to support transfer by helping learners abstract the key principles and features of the examples so that knowledge is not overly tied to narrow contexts (Goldstone, Day, & Son, 2010; Son, Smith, & Goldstone, 2011). Comparing two examples of a concept can highlight their shared relational structures so that learners can disregard superficial features and create generalized conceptual knowledge (Gentner & Markman, 1997). Focusing on the structural features of a concept while discounting the superficial features is theorized to support students’ ability to apply that knowledge in new examples and settings.

The benefits of comparison on many different aspects of learning are well established (Gentner & Markman, 1995; Gick & Holyoak, 1983; Loewenstein et al., 1999). Crucially, comparison promotes abstraction of generalized knowledge and transfer to novel situations (Gentner & Namy, 1999; Loewenstein et al., 1999; Thompson, Gentner, & Loewenstein, 2000). For example, learners who compare examples are more likely to describe concepts in general terms, rather than connected to the contexts of the examples (Catrambone & Holyoak, 1989; Gick & Holyoak, 1983). The benefits of comparison extend beyond the creation of generalized knowledge. Comparison promotes the understanding of both individual cases involved (Kurtz, Miao, & Gentner, 2001), produces large gains in procedural knowledge and flexibility in problem-solving (Rittle-Johnson & Star, 2007), and enhances discrimination between problem categories (Cummins, 1992; VanderStoep & Seifert, 1993). Comparison even prepares people to learn more from future direct instruction than studying individual cases (Schwartz & Bransford, 1998). Benefits of comparison have been shown across a variety of domains, including math (Ross & Kennedy, 1990), biology (Glynn & Takahashi, 1998), physics (Kurtz et al., 2001), and spatial mapping (Loewenstein & Gentner, 2001), across a wide range of ages (Gentner & Namy, 1999; Loewenstein & Gentner, 2001), and across long retention intervals (Chen & Klahr, 1999).

## Remindings

Remindings, stimulus driven retrievals of past specific episodes, allow learners to notice and identify common characteristics of related stimuli across time and distance (Benjamin & Ross, 2010). Remindings may prompt learners to recognize meaningful patterns across experiences in order to categorize new instances, generate inferences, and solve unfamiliar problems (Hintzman, 2010). Remindings may allow us to compare sequentially distant instances of a category in order to distinguish critical commonalities from irrelevant differences, generalize across events, and contrast between examples (Benjamin & Tullis, 2010). For example, when we study a new exemplar of a category, it may remind us of a previous specific instance we have seen, prompt us to identify commonalities between the stored and triggering event, and change what we believe is central to category membership (Ross, Perkins, & Tenpenny, 1990). More concretely, bird watchers may see a new bird, which reminds them of a prior bird that they have seen. The shape of their beaks may be similar, so they recognize this as an important part of the category, but their colors may differ, so the birders disregard this trait. Theories of reminding suggest that the effortful memory retrieval of the first presentation during the second presentation enhances memory for the first presentation (Tullis, Benjamin, & Ross, 2014), enables comparison between the two episodes, and fosters generalizations as a result of that comparison process (Ross & Kennedy, 1990).

Remindings are thought to support memory and a wide variety of additional higher order cognitive skills. Within memory research, remindings have been shown to enhance memory for the first instance in a related pair (Tullis, Benjamin, et al., 2014; Tullis, Braverman, Ross, & Benjamin, 2014). Further, remindings benefit recency judgments (Hintzman, 2010; Tzeng & Cotton, 1980; Winograd & Soloway, 1985), spacing judgments (Friedman & Janssen, 2010; Hintzman, Block, & Summers, 1973; Hintzman, Summers, & Block, 1975), and judgments of frequency (Hintzman, 2004). Not only do remindings benefit mnemonic performance, they also support a wide range of higher order cognitive skills, including classification of new items (Medin & Schaffer, 1978; Ross, Perkins, & Tenpenny, 1990), interpretation of ambiguous events (Ross & Bradshaw, 1994; Tullis, Braverman, et al., 2014), and generalization across episodes (Ross & Kennedy, 1990).

## Comparison versus remindings

Given the respective cognitive advantages for comparison and reminding, it is natural to wonder which process will provide greater benefits in which contexts. As a motivating example, imagine a teacher trying to teach her students about the general notion of a positive feedback loop. One possible recommendation, based on the advantages of explicit comparison, would be for her to present to students two examples of positive feedback: (1) a microphone feeding into, and placed near, a loudspeaker; and (2) children in a summer camp buying a particular brand of doll that other children in the camp had already purchased. She would then ask her students to notice the similarities between the two scenarios, with the hope that the comparison will highlight their deep similarity—the presence of an attribute in a system variable leads to further increase of the same attribute. A second possible recommendation, based on the advantages of reminding, would be for her to present one of the scenarios on Monday and the second on Tuesday, without ever juxtaposing them, with the hope that students will be reminded of Monday’s scenario when presented with Tuesday’s scenario, and once so reminded, will retrieve and strengthen the aspects of Monday’s scenario that match Tuesday’s. When are the advantages of students actively being reminded of a previous scenario sufficiently strong to justify withholding the previous scenario for direct and simultaneous comparison when the second situation is presented? Experiments 1 and 2 explore this question with respect to memory for the scenarios themselves, whereas Experiment 3 examines generalization to new examples of the principle.

Both comparison and reminding approaches ultimately suggest that benefits arise from comparing across individual episodes, but the benefits arise from different processes. Comparison de-emphasizes individual examples. Learners who are engaged in comparison are hypothesized to emphasize the structural commonalities across instances and downplay unique superficial features belonging to only example, promoting generalized knowledge. The advantages of comparison arise because learners are less likely to tie the general concepts to the particular contexts or specific episodes. Remindings, on the other hand, depend upon remembering the specific individual episodes. Learners cannot be reminded of earlier episodes if they have forgotten them (Benjamin & Tullis, 2010).

Another central difference between approaches is simultaneous versus sequential presentation of examples. The comparison process benefits from the simultaneous presentation of two distinct episodes in order to facilitate alignment between them, while reminding relies upon learners to actively retrieve the first episode from memory when triggered by the second. Invoking practice retrieval (Roediger & Karpicke, 2006) and generation effects (Slamecka & Graf, 1978) reminding theory suggests that the effortful retrieval of the first example during the presentation of the second creates the mnemonic benefits of remindings (Benjamin & Tullis, 2010). The benefits of remindings are contingent upon successful noticing and retrieval of the relevant prior knowledge during the processing of a later related episode. Successful retrieval of the earlier information is required before learners can compare and generalize across the episodes. For example, when people notice the relationship between a current item and prior information, memory for that prior information is enhanced; however, when people fail to notice that relationship, the later item can interfere with memory for the earlier item (Bellezza, Winkler, & Andrasik, 1975; Jacoby, Wahlheim, & Kelley, 2015; Wahlheim & Jacoby, 2013). Noticing and retrieving prior related information during the processing of other examples often proves to be a significant obstacle (Catrambone & Holyoak, 1990; Gick & Holyoak, 1983).

Across three experiments, we examined how comparison and remindings (1) impact memory for individual instances in related pairs and (2) foster generalized knowledge. Comparison should focus learners’ attention on the commonalities across episodes and support the creation of abstract, generalized knowledge, but may hamper memory for the individual episodes. Alternatively, if learners retrieve appropriate prior episodes during the presentation of later related episodes, remindings should benefit both memory for individual episodes and generalization across those episodes. The mnemonic and generalization consequences of comparison and remindings will be directly contrasted here.

## Experiment 1

In this first experiment, we contrasted memory for the individual episodes across a comparison group and a reminding group. Learners studied a list of proverb pairs. In the reminding condition, we prompted participants to “look back” through the studied list for a proverb that shared the same meaning as the current one, as has been done in reminding research (Jacoby et al., 2015; Wahlheim & Jacoby, 2013; Wahlheim, Maddox, & Jacoby, 2013). In the comparison condition, participants were given two proverbs and asked to compare their meaning. Insomuch as comparison fosters general, abstract knowledge over memory for the individual instances, we expect that reminding should lead to better recall performance for the instances than comparison.

## Method

### Participants

Thirty introductory psychology students at Indiana University participated in exchange for partial course credit. Thirty additional participants were tested through Amazon Mechanical Turk in exchange for $1.00. Finally, 41 participants from the University of Arizona community were recruited through fliers on campus and were paid $10 per hour of participation. The power to detect a large effect size (large effect size f = 0.25) for a three-way interaction for 101 participants is 0.98 (Cohen, 1988; GPower, 2016). The power to detect a large effect size for repeated measures ANOVA within each separate condition is 0.94 (Cohen, 1988; GPower, 2016).

### Materials

A total of 30 pairs of proverbs were gathered from Markman, Taylor, and Gentner (2007), adapted from various websites about foreign proverbs, or were created. Each proverb pair shared a deep meaning, but varied in superficial features. The proverbs are provided in the Appendix.

### Design

The experiment manipulated one variable between participants (condition: reminding versus comparison) and two variables within participants (related versus unrelated pairs and position within a pair: P1 versus P2). Participants were alternatively assigned to the reminding and comparison conditions. In the reminding condition, learners studied a single proverb at a time and were asked to type any prior studied proverb that shared the same meaning as the current one. In the comparison condition, learners viewed two proverbs simultaneously and were asked to type a comparison or generalization across the two proverbs if appropriate. Proverb pairs were randomly assigned to the related or unrelated conditions within each condition. On related trials, both proverbs with the same meaning were presented on the screen together (comparison condition) or were both presented within the study list (reminding condition). On unrelated trials, two unrelated proverbs from two different proverb pairs were randomly paired together and presented like the related pairs. Proverbs in the unrelated condition never shared their meaning with any other proverb in the study list. Finally, each proverb was randomly assigned to be presented first (P1) or second (P2) within the pair. In the comparison condition, P1s and P2s were presented on the screen together, but P1s were presented above P2s. In the reminding condition, P2s appeared 2 items after P1s.

### Procedure

The experiment was programmed using Collector software (Garcia, 2015). In this and all following experiments, participants first read and signed a consent form. Participants in the lab completed the experiment across ten different computers in individual testing booths. Participants were alternatively assigned to either the reminding or comparison conditions. Participants in the reminding condition were instructed: “You will see a series of proverbs presented one at a time. For each proverb, if you have already studied a proverb that has the same MEANING as the current one, please type it in.” If they could not think of any prior proverb that had the same meaning, they were asked to type in “None.” The next proverb was presented after the participants entered a response for the current trial. The lag between presentations of proverb pairs was one intervening item. Participants in the comparison condition were instructed that they would view two proverbs simultaneously. For each pair, they were asked to type in a comparison or generalization about the meaning of the pair of proverbs. The participants were instructed to type in “None” if they could not derive a comparison or generalization across the pair. Participants in both conditions viewed ten related and ten unrelated proverb pairs. After finishing the study phase, all participants played 3 min of Tetris. Finally, all participants engaged in a surprise free recall test, where they were asked to type in any proverbs they remembered from the study list. Participants were required to spend at least 2 min recalling proverbs before they could choose to end the recall phase.

## Results

Proverb recall was scored by three independent researchers; coders agreed on 98% of the recalled items. For the 2% of recalled items that were coded differently, scores were assigned based upon the majority of the coders. All patterns of results were similar across all three groups of participants (all ps > 0.05). Therefore, we did not differentiate between type of participants in any of our analyses.

**Fig. 1:**
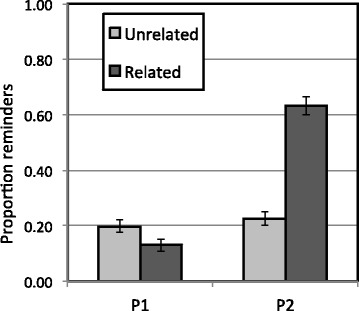
The proportion of studied proverbs that reminded learners of earlier studied proverbs in the reminding condition. *Error bars* indicate one standard error of the mean above and below the mean

To verify that later related proverbs reminded learners of previously studied proverbs, we first calculated how frequently related and unrelated P1s and P2s reminded learners of previously studied proverbs within the reminding condition. The proportion of each type of proverb that reminded learners of earlier proverbs is displayed in Fig. 1. A 2 (position) × 2 (relatedness) ANOVA on the proportion of proverbs that reminded learners of earlier proverbs showed a significant two-way interaction (F(1, 50) = 140.79, *p* < 0.001, η_p_^2^ = 0.74), a main effect of position (F(1, 50) = 176.05, *p* < 0.001, η_p_^2^ = 0.78), and a main effect of relatedness (F(1, 50) = 56.19, *p* < 0.001, η_p_^2^ = 0.53). Follow-up t-tests showed that related P2s served as reminders more frequently than unrelated P2s (t(50) = 10.83, *p* < 0.001, Cohen’s d = 1.53), but related P1s served as reminders less often than unrelated P1s (t(50) = 3.53, *p* = 0.001, Cohen’s d = 0.48). Of the related P2s that reminded participants of earlier proverbs, 69% reminded participants of the earlier proverb that shared the same deep meaning. This suggests that the relationships between proverbs supported reminders to appropriate earlier proverbs.

We also calculated how often participants in the comparison condition created a generalization compared to how often they said entered “None” because they could not create a generalization. Participants created a generalization across two proverbs more often when the proverbs were related (M = 0.94 [SD = 0.11]) than when they were unrelated (M = 0.36 [SD = 0.36]; t(49) = 10.55, *p* < 0.001, Cohen’s d = 1.51).

**Fig. 2:**
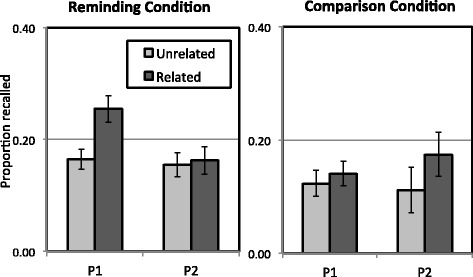
The proportion of studied proverbs recalled based upon relatedness and study position in the reminding condition (*left*) and comparison condition (*right*) in Experiment 1. *Error bars* indicate one standard error of the mean above and below the mean

The proportion of proverbs recalled is displayed in Fig. 2. A 2 (position) × 2 (relatedness) × 2 (reminding versus comparison) ANOVA on proportion recalled revealed a significant three-way interaction (F(1, 99) = 11.97, *p* = 0.001, η_p_^2^ = 0.11). The position by condition interaction reached significance (F(1,99) = 4.71, *p* = 0.03, η_p_^2^ = 0.04), but no other interactions reached significance. The ANOVA further revealed simple main effects of position (F(1, 99) = 9.61, *p* = 0.003, η_p_^2^ = 0.09) and relatedness (F(1, 99) = 13.19, *p* < 0.001, η_p_^2^ = 0.12), such that first items in a pair (P1) were remembered better than second items (P2) and related items were remembered better than unrelated items. Finally, participants in the reminding group recalled more items than those in the comparison group (F(1, 99) = 13.25, *p* < 0.001, η_p_^2^ = 0.12).

Finally, we compared the time spent per each proverb, as participants self-paced through the list of proverbs in each condition. In the comparison condition, we divided each trial time by 2 because two proverbs were presented during each trial. Participants in the compare condition spent 16.20 s [SD = 12.17] studying each proverb, while participants in the remind condition spent only 13.82 s [SD = 6.73] studying each proverb. The difference in study time between conditions did not reach significance (t(100) = 1.20, *p* = 0.23, Cohen’s d = 0.24) and was in the direction opposite that of recall performance.

## Discussion

Participants accurately connected proverb pairs that shared a deep meaning. Participants were reminded more often of prior proverbs that shared the same meaning than prior unrelated instances. Participants in the comparison condition created generalizations more frequently when the proverbs shared the same meaning. Participants understood the deep meanings of the proverbs and often relied upon them to make connections between superficially different proverbs.

Participants in the reminding condition remembered more proverbs than those in the comparison condition. Prompting learners to think back through the study list and retrieve prior related proverbs produced better memory than prompting learners to compare two proverbs. Further, participants in the reminding condition were more efficient as they recalled more items, but spent less overall time studying the proverbs. The mnemonic benefits in the reminding condition are particularly prominent for the first items in related pairs. The mnemonic advantage for related P1s over unrelated P1s replicates research using different materials and contexts and has been labeled the “reminding effect” (Tullis, Benjamin, et al., 2014). The specificity of the benefits to P1 (and not P2) likely arose because the effortful retrieval of P1 during P2 enhanced later retrieval of P1.

An alternative explanation of the benefits seen in the reminding condition is that participants may have encoded the proverbs more deeply initially and the final test reflects differential encoding practices. The results of Experiment 1 suggest that reminding instructions may contribute to the mnemonic differences between conditions, but also shows that the reminding condition entails more than just deeper initial encoding. Participants in Experiment 1 remembered more unrelated proverbs than participants in the comparison condition, which supports the idea that reminding instructions improved overall encoding. This is particularly interesting because comparison participants spent numerically more time studying the proverbs than the reminding participants. However, the specificity of mnemonic benefits to related P1s shows that reminding instructions do not prompt learners to more deeply encode all the stimuli as compared to the comparison condition. Remindings specifically benefit memory for the first instances in related pairs, which suggests that the large mnemonic consequences of remindings arise from retrieval of the first presentation during encoding of the later related episode.

We find no evidence that comparison promotes forgetting of individual instances. In fact, we find some evidence that comparing related items promotes memory for those items more than comparing unrelated items. Participants in the comparison condition mostly created generalizations across related pairs and more items from related pairs were remembered than from unrelated pairs. Comparing across similar instances seemed to enhance memory for the individual items in a comparison more than across dissimilar instances. This suggests that participants are encoding the individual instances in related pairs, even though they create a generalization. However, the mnemonic benefits of comparison are much smaller than those of reminding.

## Experiment 2

In Experiment 2, we examined memory performance after a longer retention interval in order to assess whether the mnemonic advantage of reminding over comparison persists over time. Participants completed a recognition memory test one week after they studied the sequence of proverbs. Further, the recognition test was designed to assess whether comparison participants remembered the general principles of proverbs, but forgot the specific episodes. The recognition test in Experiment 2 included proverbs that were never studied but shared the same meaning as a pair of studied proverbs. In Experiment 1, the reminding group may have benefited because their encoding phase was very similar to their testing phase: they typed in specific proverbs during both study and test. Experiment 2 suffers less from this concern because the final memory test involved recognition (and not recall), where learners did not type in any proverbs and which likely relied upon different aspects of memory than recall.

## Methods

### Participants

Eighty participants from Amazon Mechanical Turk completed the first part of the experiment for $0.75. Participants were contacted one week later and asked to complete the second part of the experiment for $1.25. Sixty-nine participants finished the second part of the experiment. We only analyzed data from the 69 participants who completed both portions of the experiment in our analyses. Our power to detect large sized effects (effect size d = 0.8) using two-tailed t-tests between the two conditions (Cohen, 1988; GPower, 2016) with 69 participants is 0.93. For repeated measures t-tests within each condition, our power to detect large effects is 0.96.

### Materials

The 30 pairs of proverbs from Experiment 1 were expanded to 40 triplets of proverbs. Forty triplets of proverbs were gathered from various websites, books of foreign translations of proverbs, or were created. Each triplet shared a similar deep meaning, but varied in superficial features. Six proverbs unrelated to any of the triplets were also gathered to be used as filler items.

### Design

Participants were randomly assigned to reminding and comparison conditions. The study phase was similar to Experiment 1, but the composition of the study list differed. Participants studied 20 pairs of related proverbs and three pairs of unrelated filler items. Proverb triplets were randomly assigned to be studied or unstudied. For the studied triplets, two out of three proverbs were randomly selected to be studied and the study order of the two studied proverbs was randomized. As in Experiment 1, proverb pairs in the reminding condition were separated by one intervening proverb. The position of each proverb pair within the study list was random.

The testing procedure differed largely from Experiment 1. Learners were given a recognition test that included ten studied P1 proverbs (studied items), ten unstudied proverbs that shared the same deep meaning as a studied pair (unstudied, but related), and 20 unstudied proverbs that were not related to any studied proverb (unstudied and unrelated). One proverb from each unstudied triplet was randomly chosen to be on the list of the 20 unstudied, unrelated proverbs. Only studied P1s (and not studied P2s) were included within the recognition test because presenting proverbs to participants during a recognition test may impact their later recognition of related proverbs (Tullis, Benjamin, et al., 2014).

### Procedure

The study phase was very similar to Experiment 1, except that only three out of the 23 studied pairs of proverbs were unrelated. The retention interval was increased to one week to assess the persistence of mnemonic benefits. Participants were contacted through email one week after they completed the study phase and were asked to complete the final recognition test to earn an additional $1.25. During the recognition test, proverbs were presented one at a time and participants judged them as either “studied” or “unstudied” from the first part of the experiment.

## Results

First, we analyzed how likely P1s and P2s reminded learners of earlier studied proverbs for the reminding group of participants. P2s were more likely to remind learners of a previous proverb (M = 0.66 [SD = 0.22]) than were P1s (M = 0.11 [SD = 0.10]; t(33) = 12.96, *p* < 0.001, Cohen’s d = 2.26). Of the P2s that served as reminders, 85% reminded participants of the earlier proverb that shared the same deep meaning. For participants in the comparison condition, we calculated how often they created a generalization across the related items and across the unrelated filler pairs. Participants generalized across 84% (SD = 0.24) of the related proverb pairs, but only 19% (SD = 0.26) of the unrelated filler pairs.

**Fig. 3:**
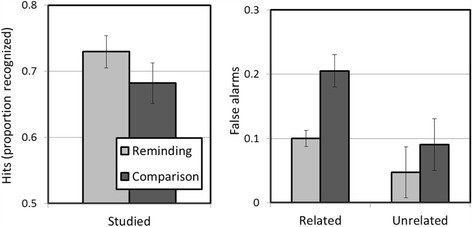
The proportion of proverbs that participants endorsed as having been studied in Experiment 2. Hits are shown on in the *left panel* and false alarms are shown on the *right. Error bars* indicate one standard error of the mean above and below the mean

The proportion of proverbs that participants endorsed as having been studied is displayed in Fig. 3. Participants in the reminding condition had fewer false alarms to unstudied, but related proverbs than participants in the comparison condition (t(67) = 2.62, *p* = 0.01, Cohen’s d = 0.68), but there were no significant differences in false alarms to unstudied, unrelated proverbs (t(67) = 1.50, *p* = 0.14, Cohen’s d = 0.37) or in hits (t(67) = 0.83, *p* = 0.41, Cohen’s d = 0.21). We combined the hits to studied proverbs and false alarms to the unstudied, but related proverbs into signal detection theoretic measures of memory performance (d’ and C; Green & Swets, 1966). Floor and ceiling performance was corrected by adding or subtracting half an item (Green & Swets, 1966). Participants in the reminding condition had greater discrimination between studied and unstudied, related items (M = 2.00 [SD = 0.77]) than participants in the comparison condition (M = 1.45 [SD = 0.91]; t(67) = 2.64, *p* = 0.01, Cohen’s d = 0.65). No significant differences were found between the criteria used to endorse proverbs between the reminding group (M = 0.28 [SD = 0.49]) and comparison group (M = 0.19 [SD = 0.48]; t(67) = 0.79, *p* = 0.43, Cohen’s D = 0.19).

Finally, we compared the time each participant spent studying each proverb. As in Experiment 1, participants in the compare condition spent 23.56 s [SD = 20.10] per proverb, while participants in the remind condition spent only 17.64 s [SD = 10.60] per proverb. The difference in study time between conditions did not reach significance (t(67) = 1.50, *p* = 0.14, Cohen’s d = 0.37) and was in the direction opposite that of final recall performance.

## Discussion

Experiment 2 replicated and extended Experiment 1. During encoding, learners in the reminding group were able to successfully connect two superficially different proverbs that shared the same deep meaning, as indicated by the high rate with which P2 reminded learners of P1. Further, reminding led to better memory performance than comparison, even after a one-week retention interval. Participants in the reminding condition were better able to discriminate between studied and unstudied proverbs than participants in the comparison condition. Comparison led to lower hit rates of studied proverbs and higher false alarms to all unstudied proverbs. Participants in the reminding condition were also more efficient at their memory processes; they spent less time but had better recognition performance than those in the comparison condition.

If comparison created abstracted knowledge about the principles put forth by proverb pairs, participants in the comparison group would have shown selectively higher false alarms to unstudied but related proverbs than the reminding group. Some evidence supports this hypothesis. A 2 (condition) × 2 (type of false alarm) ANOVA on proportion of false alarms revealed a significant interaction (F(1, 67) = 4.42, *p* = 0.04, η_p_^2^ = 0.06) and main effects of condition (F(1, 67) = 5.50, *p* = 0.02, η_p_^2^ = 0.07) and type of false alarm (F(1, 67) = 32.46, *p* < 0.01, η_p_^2^ = 0.33). The interaction indicates that comparison produced selectively more false alarms to unstudied, but related proverbs than did reminding. Comparison, then, may promote memory for generalizations of meaning across proverbs at the expense of memory for individual instances. However, we must be cautious with the interpretation of these data because scaling issues (i.e. floor effects) may be driving this interaction.

The results of Experiments 1 and 2 show that reminding supports better memory performance than comparison. In the reminding group, learners had to actively think back and retrieve prior related episodes. Remindings prompted learners to practice retrieval of a prior episode at a time when the prior episode could be recalled, re-exposed learners to the earlier episode, and supported long-term retention of that information. Comparison may have emphasized generalization at the expense of memory for the specific episodes. Results seem to suggest that participants in the comparison condition focused on abstracting a generalization from the two proverbs without encoding each proverb deeply.

## Experiment 3

Comparison is thought to primarily benefit generalization and transfer, while the benefits of reminding may primarily involve verbatim memory. Indeed, Experiments 1 and 2 showed that reminding promoted better memory performance for individual episodes than comparison. In Experiment 3, we extended beyond basic memory tasks and examined how well reminding and comparison supported transfer to new items sharing deep meanings with studied items. Transfer is thought to be one of the primary benefits of comparison. However, remindings are also thought to promote transfer, as a learner retrieves the prior episode when prompted by a later episode, compares the two, and generalizes across them. Further, practice retrieval can support performance on near transfer tasks (Butler, 2010; Rohrer, Taylor, & Sholar, 2010). During the test in Experiment 3, participants were presented with proverbs that were not studied and were asked to identify those that shared a meaning with any studied proverb. We compared the benefits of remindings and comparison in this near transfer task, as learners used the memory for the principle of studied proverbs to recognize and classify new, unstudied proverbs (Barnett & Ceci, 2002).

## Methods

### Participants

Forty-six participants from Amazon Mechanical Turk completed the experiment for $1.00. The power to detect large effect sizes (effect size = 0.8) between the conditions with two-tailed t-tests for 46 participants is 0.87 (Cohen, 1988; GPower, 2016). The power to detect large effect sizes from repeated measures within each condition with two-tailed t-tests is 0.90.

### Materials

The same 40 triplets of proverbs from Experiment 2 were used.

### Procedure

Participants were randomly assigned to the reminding and comparison conditions. The study phase was identical to Experiment 2, but the test phase differed. After the study phase, participants played Tetris for 3 min. Then, participants were presented with a list of 40 unstudied proverbs one at a time. Half of the proverbs in the test list shared a deep meaning with a pair that had been studied and half were unrelated to any that had been studied. Participants were asked to endorse any proverbs that shared the same meaning as any of the proverbs that they studied.

## Results

We first analyzed the proportion of P1s and P2s that reminded participants of earlier proverbs within the reminding condition. P2s reminded participants of prior proverbs more often (M = 0.73 [SD = 0.22]) than P1s (M = 0.08 [SD = 0.07]; t(21) = 14.94, *p* < 0.001, Cohen’s d = 3.26). Of the P2s that reminded participants of earlier proverbs, 94% reminded participants of the earlier proverb that shared the same deep meaning. We calculated how often participants in the comparison condition created a generalization across the related proverb pairs and across the unrelated filler pairs. Participants generalized across 89% (SD = 0.15) of the related proverb pairs, but only 35% (SD = 0.34) of the unrelated filler pairs.

**Fig. 4:**
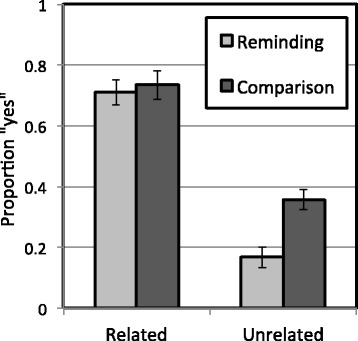
The proportion of related and unrelated proverbs endorsed in Experiment 3. *Error bars* indicate one standard error of the mean above and below the mean

The proportion of proverbs that participants endorsed as being related to ones studied is displayed in Fig. 4. Participants in the reminding condition endorsed fewer unrelated proverbs than participants in the comparison condition (t(42) = 3.96, *p* < 0.001, Cohen’s d = 1.19), but there were no differences in the endorsement of proverbs related to studied ones (t(42) = 0.38, *p* = 0.71, Cohen’s d = 0.09). We computed signal detection theoretic measures of discrimination and bias in participants’ responses by considering “yes” responses to related proverbs to be hits and “yes” responses to unrelated proverbs to be false alarms (Green & Swets, 1966). Participants in the reminding group better discriminated between related and unrelated proverbs (M = 1.53 [SD = 0.80]) than those in the comparison group (M = 1.00 [SD = 0.85]; t(44) = 2.11, *p* = 0.04, Cohen’s d = 0.65). Further, participants in the reminding group had a more conservative criterion (M = 0.24 [SD = 0.40]) than those in the comparison group (M = −0.16 [SD = 0.47]; t(44) = 3.01, *p* = 0.004, Cohen’s d = 0.92).

Finally, we analyzed the time devoted to studying each proverb in each condition. As in the prior two experiments, participants in the reminding condition spent less time (M = 16.16 [SD = 7.26]) per proverb than participants in the comparison condition (M = 19.00 [SD = 12.24]), but this difference did not reach significance (t(44) = 0.36, *p* = 0.92, Cohen’s d = 0.28).

## Discussion

Participants in the reminding condition more appropriately generalized and transferred their experiences to a new situation than participants in the comparison group. Comparison did not increase the hit rate to proverbs related to studied items, but almost doubled the false alarm rate to items that were not related to studied items. Consequently, comparison resulted in worse discrimination between proverbs that were related to studied items and proverbs that were unrelated to studied items. Comparison also resulted in a more liberal criterion for participants’ endorsement. Comparison did not support participants’ encoding of the studied principles or examples and seems to have prompted learners to overgeneralize the abstractions they created during the encoding phase. Reminding led to better transfer of studied principles to new situations and contexts than comparison. Participants in the reminding performed better on the transfer task than participants in the comparison condition. This is especially surprising because the study and test conditions were more similar for the comparison group than the reminding group: participants in the comparison group typed in themes during study and identified old proverb themes during the final test, while participants in the reminding condition typed in specific proverbs during study but identified old proverb themes during the final test. It is compelling that participants in the comparison group studied more similarly to how they were tested, but performed worse on the final task than participants in the reminding group.

Learners could have succeeded in the transfer task by remembering the general principles they abstracted from individual instances or by remembering and generalizing an individual instance. Research suggests that learners can succeed in new situations by applying generalized abstract knowledge (Gick & Holyoak, 1983) or by applying a specific prior instance (Ross, 1984). Therefore, remembering distinct prior episodes may be advantageous when trying to transfer knowledge to a new situation, as long as those prior episodes are not too tightly connected to the prior context. Reminding through structural similarities may promote both memory for the individual instances and the general principles that connect different episodes. Participants must abstract the deep meaning of the proverbs to create connections between them and the retrieval of the earlier episode during later presentations likely supports memory for the instances and the generalization. These results suggest that, relative to reminding, comparison may only support immediate generalization and not long-term memory or transfer performance. The generalization that participants create during comparison (and the individual instances compared) may be forgotten more quickly than during reminding so that participants perform poorly on later memory and transfer tasks.

## General discussion

Across three experiments, we compared the mnemonic and transfer consequences of comparison with those of reminding. In the first two experiments, reminding led to better memory for the individual instances than comparison. However, Experiments 1 and 2 suffer from the limitation that learners in the reminding group may have been using similar mental processes involving memory during both encoding and test. Because the mental processes during study and test may be more similar for the reminding group than comparison group, it may not be surprising that encoding through reminding is more beneficial for memory than encoding through comparison. In the last experiment, though, reminding showed cognitive benefits beyond just pure memory. Experiment 3 showed that reminding led to better generalization and transfer of that knowledge to a new situation. Learners who were in the reminding group more appropriately recognized proverbs that shared the same meaning as ones they had studied. Reminding not only supported memory, but also fostered appropriate transfer to new situations.

Comparison has been described as one of the most effective techniques to create generalized, transferrable knowledge (Gentner & Markman, 1997; Hummel & Holyoak, 1997). Yet, here we show that remindings led to better transfer than comparison. Our reminding task employs several well-documented cognitive principles to result in the mnemonic and transfer benefits showcased across the three experiments. First, we prompted learners to actively think back to proverbs that shared similar structural meanings with the current proverb, rather than earlier proverbs with similar superficial features. This likely encouraged learners to encode each proverb according to its deep meaning so that they could make connections between the current instance and future structurally similar instances. Encoding the proverbs in structural ways makes learners more likely to be able to later retrieve the example when they encounter a later analogous item (Loewenstein, 2010). Further, telling learners that prior instances are related to current items dramatically increases how much learners use prior examples to solve current ones (Gick & Holyoak, 1983). By giving learners a “hint” to look back through the study list, we likely increased the number of connections made between superficially different but deeply related proverbs. As learners look back through the study list, they bring together physically and temporally disparate events, the events become mentally contiguous, and learners can generalize across them.

Second, the reminding procedure forced learners to effortfully think back to and retrieve similar instances. The effortful retrieval of earlier episodes largely benefits memory for the retrieved episode (Roediger & Karpicke, 2006). The mnemonic benefits, especially for related P1s in the reminding condition, are likely due to practice retrieval. The presentation of a related P2 allows learners to practice retrieving P1 before P1 is completely forgotten. Successful practice retrieval of P1 ensures that memory for that prior item will persist over longer periods of time (Roediger & Karpicke, 2006). Practice retrieving has even been shown to benefit transfer of knowledge to new situations, tests, and contexts (Butler, 2010; Jacoby, Wahlheim, & Coane, 2010; Rohrer et al., 2010). Remindings, then, likely engender practice retrieval and, therefore, support learners’ memories of the exact studied items and learners’ generalization of studied information to new situations. Further, the separation of instances required for remindings likely takes advantage of the spacing effect, whereby repetitions presented spaced apart in time are remembered better than repetitions presented massed together (Benjamin & Tullis, 2010; Hintzman, 1974).

Third, our reminding procedure involved interleaving exemplars from different pairs. In the reminding condition, the structurally related pairs were separated by an intervening item from a structurally different pair. Interleaving exemplars from different categories can lead to enhanced memory for the items and superior category learning (Kornell & Bjork, 2008; Rawson, Thomas, & Jacoby, 2015). The benefits of interleaving exemplars from different categories is thought to arise because learners can better discriminate among categories when they are intermixed (Birnbaum, Kornell, Bjork, & Bjork, 2013; Kang & Pashler, 2012) to the degree that the categories are similar to each other (Carvalho & Goldstone, 2014). Reminding intermixes stimuli so that learners can compare and contrast between different pairs of proverbs, while the comparison process reduces the likelihood that learners compare across pairs. Interleaving, then, may cause the generalizations that arise through the reminding process to be appropriately broad.

Making physically separate events mentally contiguous through controlled memory search may be important to form complex memory traces involving both events (Jacoby, 1974). Hintzman (2011) argues that these complex memory traces incorporate the earlier stimuli into the representations of the later episode through recursive reminding and this incorporation results in the mnemonic benefits seen for related items. However, making related proverbs contiguous cannot be the fundamental cause of the benefits of reminding. Related proverbs in the comparison condition were both mentally and physically contiguous, yet were not remembered as well or transferred as appropriately. It seems likely, then, that the benefits of the practice retrieval that enables events to become mentally contiguous underlies the significant mnemonic benefits apparent in remindings.

Remindings may be most beneficial when learners can extract the deep structure of episodes and connect across superficially different instances. Comparison may be more beneficial in situations where learners need more explicit prompting to connect across very different individual instances. Further, comparison may be more beneficial with complex stimuli. In most applications of comparison during learning, the instances are presented simultaneously, so the learner does not need to occupy working memory with the retrieval of a prior episode during the presentation of a second. However, in remindings, learners need to retrieve the prior instance into working memory during presentation of the second item. Remindings, then, may require more working memory than comparison. As the to-be-learned information becomes more complex, space in working memory is likely at a premium and remindings may require more working memory than is available.

Many factors varied across the reminding and comparison conditions. Alternative experiments could have more rigidly controlled the similarities between the two conditions to isolate the factors causing differences between the conditions; however, controlling for those differences obscures the naturalistic integrity of the two conditions for real-world applications and implementation. For example, related proverbs in the reminding condition were separated by intervening unrelated proverbs while related proverbs in the comparison were simultaneously presented. Attempting to control for the simultaneous versus sequential presentation of exemplars would weaken the external validity of our procedures. The conditions we selected help answer the real-world question: if you have a limited amount of time to study related material, should you capitalize on the benefits of reminding or comparison? These results suggest that the benefits of reminding can outweigh those of comparison, at least for these stimuli.

How well the mnemonic and generalization benefits of reminding apply to different category structures and more complex materials, like complicated physics problems, remains unknown. Learners, especially novices, do retrieve and use prior problems when attempting to solve novel problems (Pirolli & Anderson, 1985; Reed, Dempster, & Ettinger, 1985). Learners need to recognize the deep structure of the problem before they can connect current problems with structurally similar prior problems. Without understanding structural similarities, learners often connect superficially similar problems that may not share deep structure (Ross, 1984). Further, the kind of information that is recalled from the prior problem during the presentation of the second likely changes what information is ultimately remembered and generalized. If learners primarily recall superficial features, without accessing deep structure, only memory for those superficial features may be enhanced. Ross (1987), however, suggests that remindings across problems can support access to the formula used to solve the prior problem. If an appropriate reminding supports retrieval of the formula, memory for that formula would likely be strongly enhanced.

The relationship between related exemplars and among the different related pairs may also moderate the advantages of remindings over comparison. Category learning research has examined how with-in and between-category similarity affects the advantages of comparison. Learners benefit from actively contrasting between confusable categories, but do not benefit much from contrasting distinct categories (Carvalho & Goldstone, 2014; Kornell & Bjork, 2008). For example, presenting two exemplars from confusable categories simultaneously can support memory for those exemplars and classification of later novel category members because it enables learners to discriminate between categories (Wahlheim, Dunlosky, & Jacoby, 2011). Here, however, we may find no mnemonic advantage of presenting exemplars from different categories together on the screen, as compared to presenting exemplars from the same category together or to presenting exemplars sequentially. The structure of our categories (each pair of related proverbs) may be low in with-in category similarity but high in between-category discriminability, such that comparison does not benefit category learning. Further, the structure of categories may determine the relative effectiveness of comparison and reminding. The benefits of remindings are likely limited by the probability of being reminded across instances. If categories have low within-category similarity, remindings may be very unlikely to occur and comparison may result in better learning and transfer than remindings.

Reminding may prove to be a desirable difficulty (Schmidt & Bjork, 1992), as compared to comparison. Comparison may allow for learners to abstract general principles from individual instances without much mental effort. With reminding, learners have to effortfully retrieve a prior related instance during the presentation of a temporally distant instance. This effortful retrieval supports memory for the first instance in a related pair (Roediger & Karpicke, 2006; Tullis, Benjamin, et al., 2014). Further, retrieving the first instance during the presentation of the second allows the learner to compare across the instances and create generalized knowledge across the episodes. Remindings seem to both enhance memory for the first presentation and generate abstracted knowledge across presentations and thereby influence both what is learned and how well it is learned.

## Conclusions

Comparison and remindings are both important cognitive processes that allow us to generalize across similar instances and generate new, abstracted knowledge. Remindings inherently involve a comparison process. When learners bring two episodes together in their mind, they compare across them and attempt to create generalized knowledge. While both comparison and reminding involve connecting related episodes, remindings impose a desirable memory requirement about individual instances on learners that comparison does not. The current experiments indicate that the memory requirement of reminding (i.e. retrieving the first presentation at the time of the second) produces large mnemonic benefits for the individual instances that can even support generalization of instances to new, superficially dissimilar but deeply related instances.

### Additional file

Additional file 1: Raw data from Experiments 1-3. (XLSX 2704 kb)

## Appendix

**Table 1:** Examples of proverbs used throughout the experiments. Each row shares a deep meaning. In Experiment 1, only the left and middle columns were used. In Experiments 2 & 3, all three proverbs were used

Even fools stumble upon insight occasionally.	Blind squirrels can sometimes find an acorn.	A broken clock is right twice a day.
A bad workman blames his tools.	An uncoordinated dancer accuses an uneven floor.	A poor rower points to the oar.
Two captains sink the ship.	Into ruin falls a village with many mayors.	Too many midwives deliver a sickly baby.
Don’t make clothes before the baby is born.	Don’t heat the oil until the fish is on the bank.	It’s not wheat until it’s been harvested.
To eat walnuts, you must crack some shells.	Woodchips must fly when you cut wood.	You can’t make an omelet without breaking some eggs.
The leopard can’t change his spots.	A pig will always oink and never purr.	A rose is a rose is a rose is a rose.
Drops gather one at a time to become an ocean.	Pennies and pennies make pounds and pounds.	Hair by hair the head goes bald.
The fat man thinks no one is hungry.	A liar expects everyone to lie.	If you sit in a hot bath, you believe the whole town is warm.
One key does not open every lock.	No single plant grows in every soil.	One instrument cannot play all the parts.
Beer will quench your thirst if the wine barrels run dry.	If the chair is broken, sit on the footstool.	Cake will satisfy when the pie is gone.
Nothing ventured, nothing gained.	If you do not enter the tiger’s den, you cannot catch its cub.	No sweet without sweat.
It’s too late to cover the well when the child is drowned.	Beating the drum is in vain after the party has ended.	Closing the barn door is pointless once the horse has escaped.
He who has once burnt his mouth will forever blow on his soup.	Bitten by a snake, afraid of the rope for ten years.	A burnt child dreads fire.
As you make your bed, so must you lie in it.	You shall harvest as you plant.	The forest echoes back just as you call into it.
I escaped the thunder but fell into lightning.	Out of the frying pan, but into the fire.	In fleeing the wolf, I ran into the bear.
Good knives cannot be forged from weak steel.	Bad flour does not make good bread.	You can’t make a silk purse out of a sow’s ear.
There are no fields without stones.	Honey is sweet, but the bees sting.	If you play with the cat, you must not mind her scratch.
A monkey in silk is a monkey no less.	A book with a fancy cover reads no better.	A broom bound with silk sweeps as well as one bound with string.
There’s no hearth like your own hearth.	Dry bread at home is better than roast meat abroad.	East or west, home is best.
Dip from the well needlessly and it will go dry.	Milk a cow too often and you will draw blood.	Load too much into a bag and it will rip.
The branch that pokes out will be trimmed.	The nail that sticks up will be hammered down.	The tallest blade of grass is the first to be cut.
One rotten apple spoils the whole barrel.	One bit of rat dung in the soup ruins the pot.	A spoonful of tar wrecks the entire jar of honey.
Different strokes suit different folks.	One man’s meat is another man’s poison.	Trash to one is treasure to another.
He who sits next to a snake begins to slither.	Those who sleep with dogs get fleas.	The friend of a thief becomes a thief.
An empty vessel makes the most noise.	It is not the sheep that baas the most that gives the most wool.	The hen that cackles loudest doesn’t always lay the best eggs.
Even expensive silver will eventually tarnish.	No rose stays red for a hundred days.	All good things must come to an end.
The shoemaker’s children often go barefoot.	The smith’s horse is the worst shod.	The man in town with the worst haircut is the barber.
The strength of the seaman make the captain succeed.	The servant’s toil makes the master look good.	The guts of the soldier make the general great.
Who holds two watermelons in one hand will drop both.	Going after two hares at the same time will allow the hares to escape.	He with too many irons in the fire will ruin them all.
Bigger roosters start to crow later.	Larger pots boil slower.	Greater insights take longer to discover.
